# Terminology of pleural artifacts in ultrasound: a Tower of Babel

**DOI:** 10.1186/s13244-026-02234-3

**Published:** 2026-03-12

**Authors:** Ehsan Safai Zadeh, Libertario Demi, Christian Görg, Veronika Vetchy, Felix Ragnar Merlin Koenig, Michael Weber, Pascal Baltzer, Helmut Prosch

**Affiliations:** 1 Department of Biomedical Imaging and Image-guided Therapy, Medical University of Vienna, Wien, Austria; 2 Department of Information Engineering and Computer Science, University of Trento, Trento, Italy; 3 Interdisciplinary Center of Ultrasound Diagnostics, Gastroenterology, Endocrinology, Metabolism and Clinical Infectiology, University Hospital Giessen and Marburg, Philipps University Marburg, Marburg, Germany

**Keywords:** Lung ultrasound, Pleural artifacts, Ultrasound terminology, Standardized nomenclature

## Abstract

**Objectives:**

Lung ultrasound (LUS) has evolved into a widely used tool for the assessment of pleural artifacts (PA), yet the field remains trapped in a terminology Tower of Babel. A proliferation of terms has fragmented the literature, obstructed communication, and hindered progress toward standardization.

**Materials and methods:**

Between January and February 2025, a structured but unsystematic literature review was conducted via Google Scholar to identify terminology used for PA. Twelve distinct terms were identified: A-lines, B-lines, comet-tail artifact, vertical artifact, horizontal artifact, reverberation artifact, mirror-image artifact, ring-down artifact, interstitial syndrome, wet lung, dry lung, and aurora sign. Relevant publications were selected based on predefined keyword combinations and expert review. An analysis of terminology usage was performed for 2000–2025.

**Results:**

The analysis revealed an increase in PA mentions from 751 (2000–2004) to 16,269 (2020–2025), representing more than a twenty-fold increase (+2,066%). Eleven of the twelve PAs showed significant annual growth; only the aurora sign did not demonstrate a consistent increase in usage over time.

**Conclusion:**

The simultaneous increase in nearly all terms for the same physical phenomena reflects a persistent lack of consensus in the literature. While other imaging fields, such as computed tomography, have adopted standardized glossaries like that of the Fleischner Society, LUS remains without a unified terminology. A shared, technology-independent vocabulary is needed to ensure clarity, comparability, and future progress in LUS.

**Critical relevance statement:**

A shared, technology-independent vocabulary is needed to ensure clarity, comparability, and future progress in lung ultrasound (LUS).

**Key Points:**

- Standardized studies and methodological progress require a unified nomenclature.
- The ultrasound community currently lacks consistent terminology for pleural artifacts (PA).
- A consensus, unified terminology for PA is urgently needed.

**Graphical Abstract:**

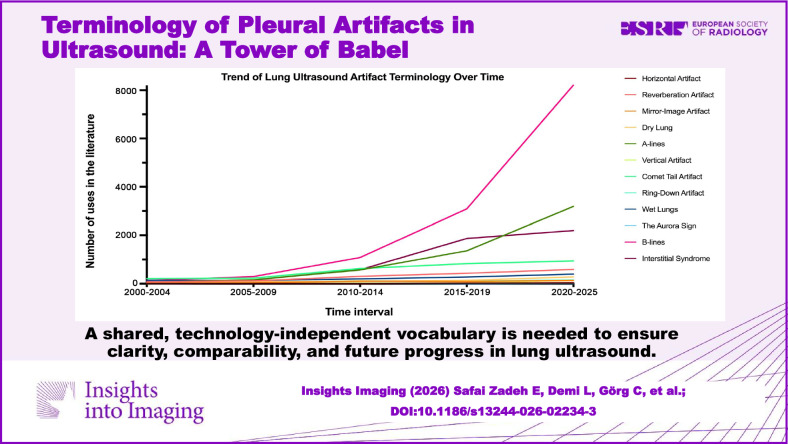

## Introduction

According to the biblical narrative, after the great flood, people decided to build a city with a tower that would reach into the heavens, “the Tower of Babel.” God observed the human endeavor and decided to confuse their languages, making it impossible for them to communicate. As a result, the people had to cease their construction activities.

The current state in the lung ultrasound (LUS) community regarding pleural artifacts (PA) is not much different. Since the introduction of LUS, various terms have been used to describe the artifacts observed. Consequently, understanding and comparing different studies is currently possible only to a limited extent. The problem of LUS terminology arose with the development of LUS and has advanced with the progress of ultrasound technology and knowledge about LUS. The potential diagnostic use of LUS was first proposed in 1946 by the French physician André Dénier, who described it as a promising method for thoracic imaging known as Ultrasonoscopie [1]. During the 1960s, research groups applied LUS, but its use remained largely limited to detecting pleural fluid [2, 3]. In the 1970s, ultrasound was successfully employed to differentiate between pulmonary consolidation and effusion [4]. However, it was in the 1980s and 1990s that continuous advancements in ultrasound imaging led to a deeper understanding and broader clinical application of LUS [5, 6]. In addition, it was recognized that artifacts observed during LUS could have diagnostic relevance [5]. The integration of artifacts into LUS interpretation not only significantly increased its clinical value but also contributed to a growing diversity of terminology. Currently, there is no standardized definition of artifacts, leading to inconsistencies in study designs, inaccurate comparisons, and potentially misleading correlations with pathological conditions. The lack of a uniform nomenclature continues to limit the comparability of studies and hinders clear interdisciplinary communication.

This study aims to analyze the evolution of LUS terminology to better understand the current challenges within the ultrasound community, to encourage acknowledgment of the issue, and to promote collaboration toward the development of a unified terminology--ultimately to enhance communication, advance LUS research and knowledge, and improve the effectiveness of clinical implementation.

## Materials and methods

Between January and February 2025, a structured but unsystematic literature review was conducted using Google Scholar to explore the terminology used for LUS artifacts from the initial application of LUS until January 2025.

Google Scholar was selected because the aim of this work was terminology mapping rather than a clinical-effectiveness systematic review. In this context, Google Scholar provides broader cross-disciplinary coverage (including non-indexed sources and books/chapters) in which LUS terminology is frequently introduced and disseminated.

No ethical approval was required for this study, as it involved only the analysis of previously published literature. Relevant publications were identified by combining keywords such as ‘lung ultrasound’ and ‘artifacts.’ Titles and abstracts were screened for relevance, and full texts were reviewed, when necessary, by a radiologist with over seven years of experience in LUS (E.S.Z.), under the supervision of an ultrasound expert certified by the Austrian Society for Ultrasound in Medicine. Selection was based on a subjective assessment of relevance and terminology use. Because no universally accepted or comprehensive list of pleural artifact terminology exists, candidate terms were identified through an initial scoping search and review of key LUS publications. Synonyms and minor spelling variations were consolidated. The final set of 12 terms was selected through expert consensus involving two independent senior thoracic ultrasound specialists (H.P. and C.G.). Table 1 summarizes the most commonly used terms found in the literature.

**Table 1:** Summary of the literature review for artifact terminology in LUS

Group	Artifact name	Definition according to the earliest descriptions in the literature
Artifacts oriented parallel to the transducer	Horizontal artifact	Regularly spaced, roughly horizontal hyperechoic lines spreading from the lung-wall interface [19, 20].
	Reverberation artifact	Reverberations between the aerated lung and the transducer surface create a characteristic periodic artifact at the interface between the aerated lung and adjacent soft-tissue structures [5].
	Mirror-image artifact	Total reflection with mirror formation at the lung-pleura interface [21].
	Dry lung	Absence of bilateral comet-tail artifacts [22].
	A-lines	Horizontal reverberation artifacts at the lung surface [7].
Artifacts oriented vertically to the transducer	Vertical artifact	Vertical comet-tail artifacts arising from the pleural line, well-defined and laser-like, moving with lung sliding, spreading to the edge of the screen without fading, and erasing normal A-lines [23–25].
	Comet-tail artifact	A trail of dense continuous echoes, simulating a comet tail [26].
	B-lines	Comet-tail artifacts originating from the pleural line, moving in synchrony with lung sliding [7].
	Interstitial syndrome	Presence of multiple diffuse bilateral B-lines [8].
	Ring-down artifact	Multiple, vertical, long, narrow bands or lines trailing down from the posterior surface of the right hemidiaphragm [27].
	Wet lung	Presence of diffuse bilateral comet-tail artifacts [22].
	Aurora sign	Ring-down artifacts observed by subcostal or intercostal US were defined as the aurora sign when they were numerous (more than 20) [28].

### Literature search strategy for the frequency of artifact terminology use

A systematic search was performed in February 2025 by a radiologist (E.S.Z.) to evaluate how often the terms for these artifacts were used over time. The following query was applied to each artifact:

“artifact name” AND (“lung” OR “thoracic” OR “thorax”) AND (“ultrasound” OR “ultrasonography” OR “echographic”)

Searches covered five intervals (2000–2004, 2005–2009, 2010–2014, 2015–2019, 2020–2025). The following criteria were assessed:

- Frequency of terminology use
- Increase in the use of different terminologies over time

### Statistical analysis

To analyze the temporal evolution of LUS artifact terminology, Poisson regression was used to model the frequency of each terminology over time. The Poisson model was chosen because the data represent count-based observations, which follow a non-negative integer distribution. Statistical analysis was performed using a version of GraphPad Prism prior to version 10.5.0. The model was structured as follows:

$${\mathrm{Log}}({{\rm{Y}}})={\beta }_{0}+{\beta }_{1}(Year)$$

where:

- Y is the observed count of a given artifact terminology in each time interval,
- ß_0_ is the intercept, and
- ß_1_ represents the yearly growth rate of terminology use.

The statistical significance of the model was evaluated using likelihood-ratio tests, and model fit was assessed using pseudo-R-squared values. A *p*-value < 0.05 was considered statistically significant.

## Results

### Frequency of different terminologies across various time periods

Over the past 25 years, mentions of LUS artifacts increased substantially across all categories, with a global rise from 751 (2000–2004) to 16,269 (2020–2025), corresponding to a 21.7-fold increase (+2066%). B-lines showed the greatest absolute increase, from 128 (2000–2004) to 8210 (2020–2025) (+6314%), accounting for 50.5% of all mentions in the final period. A-lines grew from 58 to 3190 (+5400%), Interstitial Syndrome from 34 to 2200 (+6371%), and Comet-tail Artifact from 214 to 939 (+339%). Further increases were observed for Wet Lung (137 to 395; +188%), Ring-down Artifact (23 to 156; +578%), Mirror-Image Artifact (31 to 135; +335%), Reverberation Artifact (75 to 588; +684%), Dry Lung (44 to 280; +536%), and Horizontal Artifact (4 to 38; +850%). Vertical Artifact was not observed in 2000–2004 (0 mentions) but increased to 133 in 2020–2025. The Aurora Sign showed an increase from three mentions in 2000–2004 to eight in 2005–2009 (+166.6%) and to eleven in 2010–2014 (+37.5%), followed by a decline to four mentions in 2015–2019 (−63.6%) and five mentions in 2020–2025 (+25.0%). Table 2 summarizes terminology frequencies and reports percentage changes compared to the previous period.

**Table 2:** Frequency of different terminologies used across various time periods

Year	Horizontal artifact	Reverberation artifact	Mirror-image artifact	Dry lung	A-lines	Vertical artifact	Comet-tail artifact	Ring-down artifact	Wet lung	The Aurora Sign	B-lines	Interstitial Syndrome
2000–2004	4	75	31	44	58	Not reported	214	23	137	3	128	34
2005–2009	9 (125.0%)	117 (56.0%)	38 (22.5%)	81 (84.0%)	160 (175.8%)	13	231 (7.9%)	46 (100.0%)	128 (−6.6%)	8 (166.6%)	295 (130.4%)	157 (361.7%)
2010–2014	3 (−66.7%)	304 (159.8%)	103 (171.0%)	91 (12.3%)	569 (255.6%)	20 (53.8%)	628 (171.8%)	113 (145.6%)	198 (54.6%)	11 (37.5%)	1080 (266.1%)	576 (266.8%)
2015–2019	27 (800.0%)	432 (42.1%)	90 (−12.6%)	138 (51.6%)	1360 (139.0%)	31 (55.0%)	833 (32.6%)	101 (−10.6%)	276 (39.3%)	4 (−63.6%)	3090 (186.1%)	1870 (224.6%)
2020–2025	38 (40.7%)	588 (36.1%)	135 (50.0%)	280 (102.8%)	3190 (134.5%)	133 (329.0%)	939 (12.7%)	156 (54.4%)	395 (43.1%)	5 (25.0%)	8210 (165.6%)	2200 (17.6%)

The absolute numbers indicate the frequency of each terminology used within the respective time period. The values in parentheses represent the percentage increase compared to the previous period

**Fig. 1:**
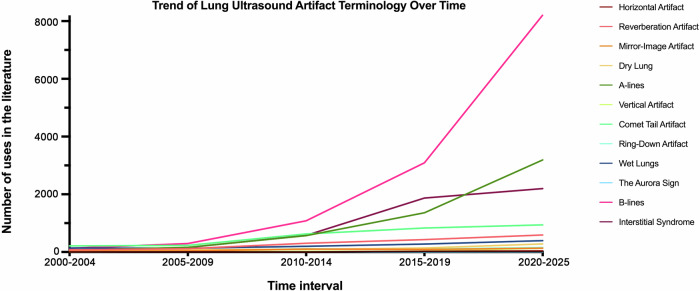
Use of different terminologies over time

The Poisson regression models quantified the rate of increase for each terminology and its statistical significance. B-lines had the strongest annual increase (β = 0.2079, *p* < 0.001), confirming an exponential rise in adoption. Vertical Artifact followed closely (β = 0.1973, *p* < 0.001). A-lines (β = 0.1865, *p* < 0.001) and Interstitial Syndrome (β = 0.1546, *p* < 0.001) showed substantial growth over time. Horizontal Artifact also increased significantly (β = 0.1225, *p* < 0.001). Reverberation artifact (β = 0.0972, *p* < 0.001), dry lung (β = 0.0906, *p* < 0.001), ring-down artifact (β = 0.0778, *p* < 0.001), comet-tail artifact (β = 0.0766, *p* < 0.001), mirror-image artifact (β = 0.0688, *p* < 0.001), and wet lung (β = 0.0609, *p* < 0.001) showed moderate but statistically significant growth. The Aurora Sign did not show a statistically significant trend (β ≈ 0, *p* = 1.000), suggesting minimal or no long-term adoption in clinical and research settings. The trend in the use of different terminologies over time is illustrated in Fig. 1.

## Discussion

The present study examines the use of LUS terminology over a 25-year period. The Year_Coefficient for all artifacts, except for the “aurora sign”—was positive and statistically significant (*p* < 0.05), indicating a consistent upward trend in use. The most significant growth was observed for “A-lines,” “B-lines,” [7] and “interstitial syndrome [8],” reflecting their widespread use. However, the continued use of multiple terms for similar phenomena highlights a persistent lack of standardized nomenclature in pleural artifact terminology.

The development of heterogeneous terminology in the description of phenomena in imaging modalities is an unavoidable issue, as medical imaging lies at the intersection of multiple disciplines and is described and evaluated from different professional perspectives. This challenge is particularly evident in ultrasound, which has traditionally been both performed and interpreted by clinicians and radiologists alike. However, it is important to understand and acknowledge the problem and to work toward a solution that ensures uniformity to improve communication between different specialties.

Although the issue of inconsistent terminology remains unresolved in the LUS community to this day, it has already been successfully addressed in other thoracic imaging modalities, such as computed tomography (CT) and chest radiography, leading to the development of the “Fleischner Society Glossary for Thoracic Imaging” [9]. The glossary highlights the importance of using standardized and precise terminology in thoracic imaging. It recognizes that effective patient care relies on close collaboration between diagnostic imaging, clinical specialties, and pathology, and that wide variability in terminology and interpretation can result in misunderstandings and inconsistent communication [10]. The glossary prioritizes the description of imaging features and patterns over specific diagnoses and favors descriptive terms over eponyms to enhance clarity and reproducibility [10]. This approach strengthens interdisciplinary communication and supports the development of clinical guidelines [10, 11]. Furthermore, standardized terminology improves the comparability of patient data and research findings, facilitates the creation of consistent datasets, supports artificial intelligence-based methods, and increases the accuracy of large-scale data analysis [11].

Several key aspects must be considered in the development of appropriate terminology. The terminology must be clear and comprehensible across all medical disciplines and usable by all involved professionals [11]. A confusing example within the LUS community, which remains unfamiliar to many clinical specialties, is the use of “A-lines” and “B-lines.” These terms originate from chest X-ray nomenclature (“Kerley A-lines” [12] and “Kerley B-lines” [12, 13]) rather than from sonographic imaging.

The term ‘Kerley B-lines’ was first introduced by James Kerley in the second edition of A Text-Book of X-Ray Diagnosis (1950) [13], describing thin, linear opacities oriented perpendicular to and abutting the lateral pleural surfaces near the lung bases [14]. In 1954, Felix George Fleischner -the namesake of the Fleischner Society- explicitly identified Kerley B lines as radiographic manifestations of thickened interlobular septa [15]. Kerley A lines, in contrast, were fine linear opacities radiating toward the hila, predominantly located in the upper lobes [14]. In 2008, after more than 50 years, the Fleischner Society recommended, in their glossary of terms, replacing the terms Kerley A and B lines with more descriptive terminology [14]. In the recently published 2024 update of the Fleischner glossary, the term ‘Kerley lines’ is classified as obsolete and no longer recommended [9].

Appropriate imaging terminology should avoid references to histopathological patterns to prevent confusion. Terms such as “interstitial lung disease,” “interstitial pattern,” or “interstitial syndrome” can be misleading in diagnostic contexts, as the underlying conditions may involve both the interstitium and the alveolar space. In addition, the term “syndrome” describes a clinical condition rather than an imaging feature [16].

**Fig. 2:**
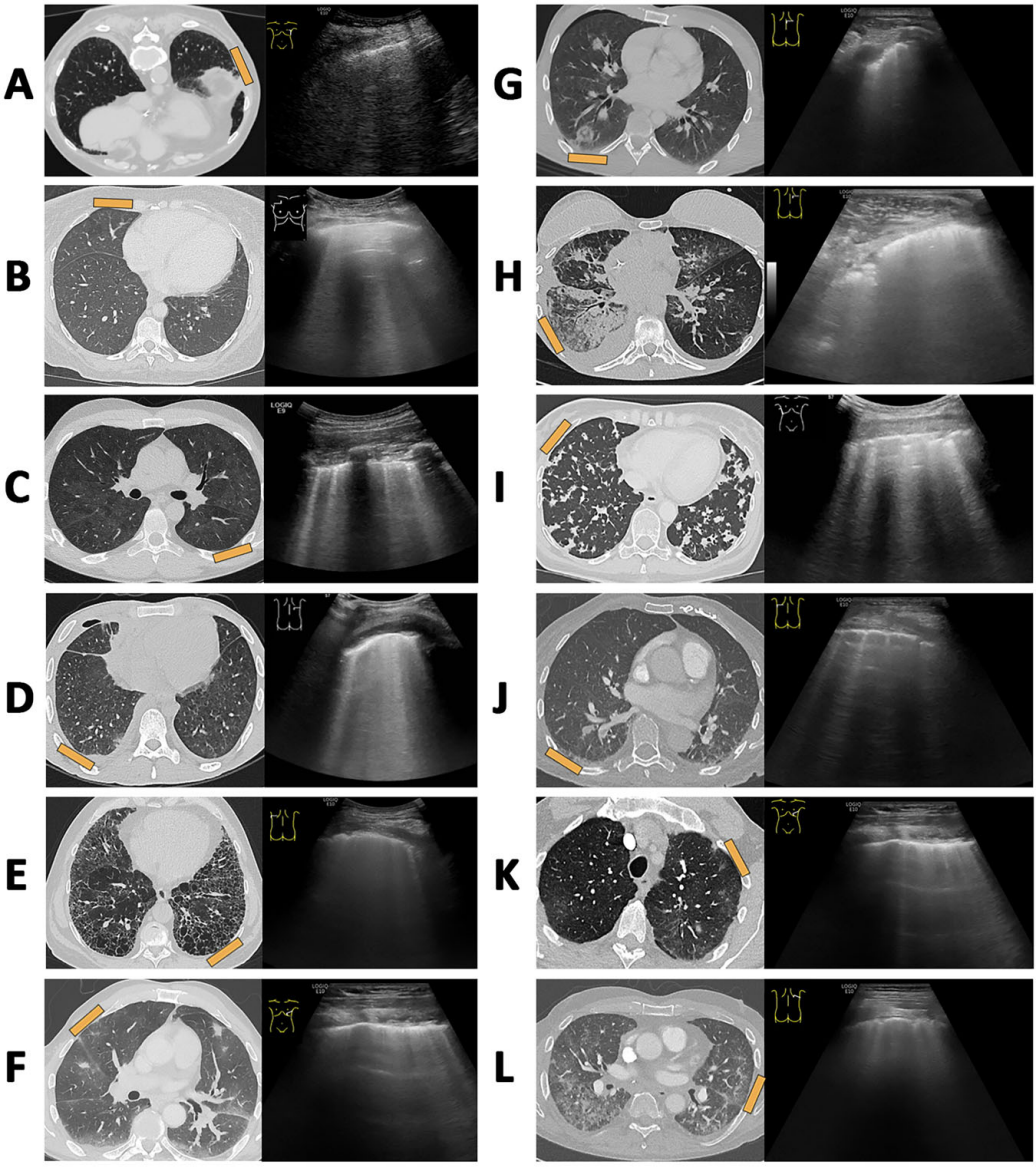
Different underlying causes of artifacts oriented vertically to the transducer in LUS. The orange marker on the CT scan (left) indicates the ultrasound probe position. **A** Patient with a history of smoking and changes caused by scarring. **B** Patient with cardiogenic pulmonary edema. **C** Patient with diffuse Pneumocystis jirovecii pneumonia. **D** Patient with pulmonary involvement in tuberculosis. **E** Patient with pulmonary fibrosis exhibiting a typical interstitial pneumonia pattern. **F** Patient with systemic sclerosis and pulmonary involvement. **G** Patient with lung contusion due to trauma. **H** Patient with breast cancer and lymphangitic carcinomatosis. **I** Patient with lepidic- to alveolar-growing carcinoma. **J** Patient with segmental pulmonary embolism. **K** Patient with pulmonary involvement in amyloidosis. **L** Patient with lung transplant rejection

Furthermore, in the development of terminology, the focus should not be on histopathological findings, as many different pathologies can present with similar imaging patterns (Fig. 2). Instead, the emphasis should be on identifying the underlying cause of the descriptive imaging pattern in the context of the patient’s clinical history, anamnesis, and, if necessary, histological findings [16].

**Fig. 3:**
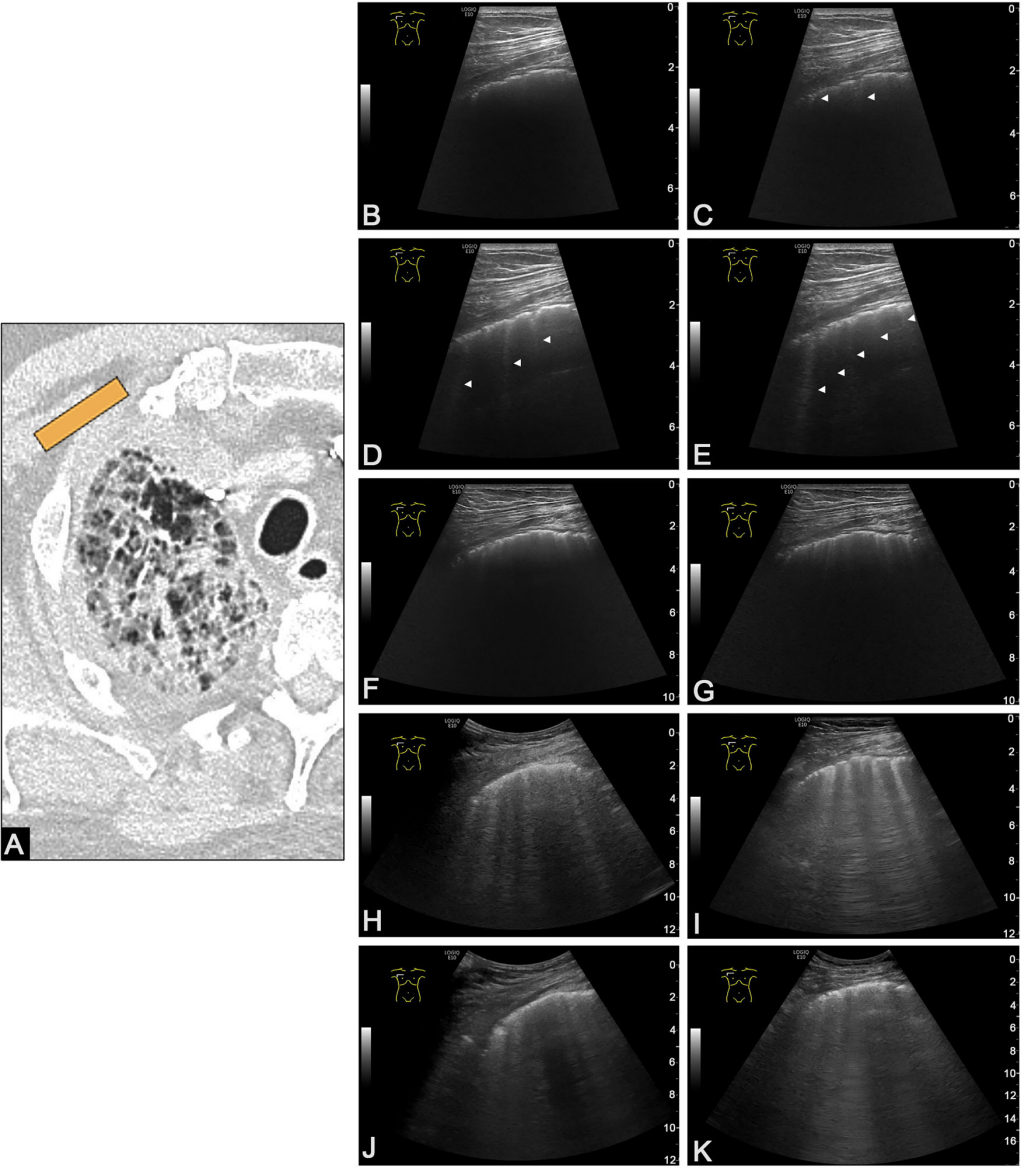
Illustration of the influence of ultrasound settings and transducer type on the number and morphology of artifacts oriented vertically to the transducer. **A** Patient with nonspecific interstitial pneumonia and pneumonia, showing ground-glass opacities and reticular parenchymal changes in the right upper lobe. The orange marker on the CT image indicates the ultrasound probe position. The same anatomical location appears differently on B-mode ultrasound, depending on the probe and machine settings. **B** High-frequency linear transducer (L8-18i) at 18 MHz with artifact suppression enabled: no artifacts oriented vertically to the transducer are visible. **C** Same transducer and frequency, artifact suppression disabled: two artifacts oriented vertically to the transducer are visible, considered non-pathological according to Lichtenstein et al [19]. **D** High-frequency linear transducer (L8-18i-RS) at 14 MHz with artifact suppression: three artifacts oriented vertically to the transducer are visible, and considered pathological according to Lichtenstein et a [19]. **E** L8-18i at 14 MHz, artifact suppression disabled: five artifacts oriented vertically to the transducer are visible, and considered pathological according to Lichtenstein et al [19]. **F** High-frequency linear transducer (ML6–15) at 15 MHz with artifact suppression: multiple artifacts oriented vertically to the transducer with triangular, tapered shape, defined as comet-tail artifacts [29]. **G** Same transducer and frequency, artifact suppression disabled: increasing number and length of artifacts oriented vertically to the transducer with triangular, tapered shape, defined as comet-tail artifacts [29]. **H** High-frequency linear transducer (9 L) at 6 MHz, artifact suppression disabled: multiple artifacts oriented vertically to the transducer, each > 10 cm in length (reaching the bottom of the image), and categorized as pathological B-lines [17]. **I** Same transducer at 5 MHz, artifact suppression disabled: increased number of artifacts oriented vertically to the transducer, all >10 cm in length, still categorized as pathological B-lines [17]. **J** Low-frequency convex transducer (C1–5) at 3 MHz, artifact suppression disabled: diffuse, barely distinguishable artifacts oriented vertically to the transducer, each > 10 cm in length, interpreted as interstitial syndrome according to Volpicelli et al [8]. **K** Same transducer at 1.5 MHz, artifact suppression disabled: extensive, confluent artifacts oriented vertically to the transducer, > 10 cm in length and indistinguishable from one another, classified as interstitial syndrome according to Volpicelli et al [8].

Finally, categorizing artifacts by their length, shape, or number should be avoided, as these characteristics are heavily influenced by technical settings and are difficult to reproduce objectively. A descriptive, technology-independent terminology would provide greater clarity and consistency. For example, the World Federation for Ultrasound in Medicine and Biology position paper defines any vertical artifact longer than 10 cm as a B-line and anything shorter as a comet-tail artifact. However, this classification is not universally applicable, as it also depends on the settings of the ultrasound system. [17]. The shape, length, and number of artifacts cannot be uniformly defined as a specific characteristic or assigned a clear clinical significance [18]. These factors depend on the ultrasound device, transducer type, and frequency, making objective evaluation difficult (Fig. 3)

## Conclusion

The primary prerequisite for advancing any technique and conducting standardized studies is the use of a unified nomenclature. This principle has long been applied in other imaging modalities, such as thoracic CT. A prominent example is the Fleischner Society glossary of terms for thoracic imaging, which is periodically updated (most recently in 2024) to ensure consistent communication among radiologists and clinicians [9]. Without this step, the ultrasound community remains trapped in the chaos of countless terminologies. Yet change is possible. The biblical account of Babel contains a reversal in the Pentecost event: while Babel led to dispersion, Pentecost brought understanding. People from diverse cultures suddenly comprehended the same message in their own languages; instead of division and confusion, Pentecost brought understanding and community.

## Data Availability

The data presented in this study are available in this article.

## Abbreviations

CT: Computed tomography
LUS: Lung ultrasound
PA: Pleural artifacts
